# The Severity of Prostaglandin-Associated Periorbitopathy Did Not Affect the Surgical Effectiveness of the Ahmed Glaucoma Valve

**DOI:** 10.3390/jcm14010042

**Published:** 2024-12-25

**Authors:** Akiko Harano, Sho Ichioka, Kana Murakami, Mizuki Iida, Masaki Tanito

**Affiliations:** Department of Ophthalmology, Shimane University Faculty of Medicine, Izumo 693-8501, Japan; ishidaki@med.shimane-u.ac.jp (A.H.); sho-ichi.1002@med.shimane-u.ac.jp (S.I.); kana1018@med.shimane-u.ac.jp (K.M.); mzk.1212@med.shimane-u.ac.jp (M.I.)

**Keywords:** surgical complication, prostaglandin FP receptor agonists, prostaglandin-associated periorbitopathy (PAP), Ahmed glaucoma valve (AGV), primary open-angle glaucoma (POAG)

## Abstract

**Introduction**: To report the role of prostaglandin-associated periorbitopathy (PAP) severity on the surgical efficacy of Ahmed Glaucoma Valve (AGV) implantation. **Subjects and Methods**: Retrospective observational case series. Participants were the consecutive 102 eyes from 102 Japanese subjects (55 males, 47 females; mean age ± standard deviation, 74.9 ± 7.8 years) who underwent AGV implantation for primary open-angle glaucoma (POAG), completed full postoperative visits for 12 months, and had information on PAP severity graded by the Shimane University PAP Grading System (SU-PAP). Data were collected via medical chart review. Comparison of surgical success rates among groups stratified by SU-PAP grades (grades 0–3) using survival curve analysis. Failure was defined based on additional glaucoma surgery, IOP reduction in less than 20%, postoperative IOP exceeding 18 mmHg (definition A) or 15 mmHg (definition B), or postoperative visual acuity reduced to no light perception. **Results**: At 12 months postoperatively, the success rates for grades 0, 1, 2, and 3 were 47%, 43%, 42%, and 73%, respectively, for definition A (*p* = 0.35) and 35%, 26%, 19%, and 27%, respectively, for definition B (*p* = 0.64, log-rank test). For definition A, younger age was associated with surgical failure (Hazard ratio = 0.97/year, *p* = 0.049, Wald test), while no other factors, including gender, preoperative IOP, medications, refractive error, history of conjunctival manipulation procedures, or SU-PAP grade, were associated with surgical failure. For definition B, no factors were found to influence surgical outcomes. **Conclusions**: The preoperative severity of PAP might not affect the postoperative outcomes of AGV. Given that the success rate of trabeculectomy is influenced by PAP severity, in cases with severe PAP, physicians are advised to consider long-tube shunt surgery as an initial filtration procedure or as a rescue procedure when filtration surgery is unsuccessful.

## 1. Introduction

Prostanoid FP receptor agonists derived from prostaglandin F2α are the primary choice for medical treatment of primary open-angle glaucoma (POAG), primarily because of their significant efficacy in reducing intraocular pressure (IOP) and their convenient dosing schedule requiring infrequent application [1]. However, this medication is associated with prostaglandin-associated periorbitopathy syndrome (PAP) [2]. PAP is known to cause a range of cosmetic changes, including superficial effects like excessive eyelash growth and eyelid pigmentation, as well as deeper tissue changes, such as deepening of the upper eyelid sulcus due to orbital fat reduction and upper eyelid ptosis caused by fibrosis of the eyelid tissue and Müller’s muscle [3].

We previously reported a unique PAP grading system called the Shimane University PAP Grading System (SU-PAP) [4]. Although other grading systems for PAP already exist [5], the SU-PAP is a novel classification method that categorizes the severity of PAP into four levels: no PAP (grade 0), superficial PAP (grade 1), deep PAP (grade 2), and tonometric PAP (grade 3). A particularly unique feature of this system is the inclusion of tonometric PAP, where the reliability of IOP measurements using Goldmann applanation tonometry (GAT) is compromised due to ptosis and severe eyelid stiffness. The SU-PAP system allows for the evaluation of not only the cosmetic changes associated with PAP but also its functional effects.

Using the SU-PAP system, the postoperative outcomes of trabeculectomy (LEC) worsen as the severity of PAP increases [6]. Miki et al. reported that the efficacy of LEC is influenced by the presence or absence of deepening of the upper eyelid sulcus (DUES) [7]. We suggested that the mechanism behind the deterioration of postoperative outcomes may involve FP agonist-induced fibrosis of the conjunctiva and the difficulty in maintaining the bleb due to compression by eyelid sclerosis. Therefore, we emphasized the importance of preventing severe and irreversible PAP in clinical settings to optimize the success of LEC. This adverse effect can be mitigated by adjusting or discontinuing the responsible medications, highlighting its avoidable nature. In patients with severe PAP, alternative surgeries such as the Ahmed Glaucoma Valve (AGV) (New World Medical, Rancho Cucamonga, CA, USA) may need to be considered.

Currently, it remains unclear whether the AGV is affected by the severity of PAP. Given that the bleb in AGV surgery is positioned farther from the limbus compared to trabeculectomy, it is possible that the AGV may be less influenced by eyelid pressure, but further investigation is required. This study evaluated how the severity of PAP influences the surgical outcomes of AGV in individuals with POAG.

## 2. Subjects and Methods

### 2.1. Study Design and Subjects

This retrospective observational case series study complied with the principles outlined in the Declaration of Helsinki. The Institutional Review Board (IRB) of Shimane University Faculty of Medicine approved the research conducted at Shimane University Hospital (IRB No. 20231018-5; approval date: 11 October 2023). Individual written informed consent for publication was waived, as the study protocol was publicly posted at the institution to inform participants. Eligible subjects were identified from the department database based on the inclusion and exclusion criteria. The inclusion criteria specified eyes that underwent AGV implantation at Shimane University Hospital between May 2018 and July 2023, diagnosed with POAG, with no history of intraocular surgeries other than uncomplicated small-incision cataract surgery, ab interno minimally invasive glaucoma procedures, or up to one prior filtering surgery (e.g., EX-PRESS or LEC). Full postoperative follow-ups were required at approximately 1 month (2–6 weeks), 3 months (1.5–4 months), 6 months (5–7 months), 9 months (8–10 months), and 12 months (11–13 months). The preoperative severity of PAP, assessed via the SU-PAP scoring system, was recorded in medical charts. Exclusion criteria encompassed eyes with conjunctival scarring over half the circumference, lens dislocation, vitreous prolapse, or complete inability to perform GAT due to PAP. If both eyes met the criteria, only the eye with the earlier surgery date was included. Ultimately, 102 eyes from 102 Japanese patients (55 men and 47 women; mean age ± standard deviation (SD), 74.9 ± 7.8 years) satisfied the criteria and were included. None of the cases were excluded due to an inability to perform GAT.

### 2.2. Surgical Procedure

At our institution, pars plana vitrectomy and AGV insertion into the pars plana are routinely performed in pseudophakic patients with posterior vitreous detachment and/or liquefied vitreous for the purpose of corneal endothelial cell preservation [8,9]. Consequently, the AGV tube was inserted into the vitreous cavity in approximately 90% of cases. To minimize variability related to surgical techniques, this study included only cases where the tube was placed in the vitreous cavity. All procedures utilized the AGV FP-7 model, which has a plate size of 184 mm^2^. The surgical procedure began with a fornix-based conjunctival peritomy and administration of sub-Tenon anesthesia using 2% lidocaine. The AGV endplate was positioned in a subconjunctival pocket between the two rectus muscles and anchored to the scleral surface with two 5–0 polyester sutures, approximately 8.5 mm posterior to the corneal limbus. For tube placement, a 25-gauge needle was used to create a scleral tunnel under a half-thickness rectangular scleral flap, positioned 3.5 mm posterior to the surgical limbus. The tube was trimmed, inserted into the vitreous cavity, and covered by closing the scleral flap with interrupted 10–0 nylon sutures. Vitrectomy was performed prior to tube insertion using a pars plana vitrectomy system to remove residual vitreous. The conjunctiva was closed with 10–0 polyglactin sutures. To reduce the likelihood of a hypertensive phase, 15 mg of triamcinolone acetonide was injected around the AGV plate at the end of surgery. Mitomycin C was not used during the surgery. Postoperative care included topical administration of 1.5% levofloxacin and 0.1% betamethasone for one month in all cases.

### 2.3. SU-PAP

The SU-PAP grading system classifies PAP severity into four levels, considering both its physical presentation and its impact on the reliability of GAT performance [4]. The grading criteria were designed based on the mechanisms underlying each component of PAP, as previously outlined.

- **Grade 0 (no PAP):** Absence of prostaglandin-associated cosmetic changes visible through macroscopic or slit-lamp examination.
- **Grade 1 (superficial cosmetic PAP):** Presence of eyelid hyperpigmentation and/or increased eyelash growth.
- **Grade 2 (deep cosmetic PAP):** Involvement of at least one feature such as DUES, blepharochalasis involution, periorbital fat atrophy, or enophthalmos.
- **Grade 3 (tonometric PAP):** Difficulty in performing GAT or reduced reliability of GAT due to PAP-related factors, including DUES, eyelid hardening, ptosis, or enophthalmos.

The subjective judgment of examiners was used to evaluate the difficulty or decreased reliability associated with GAT in Grade 3 cases. To assess the consistency of the SU-PAP grading system, inter-grader agreement between two observers (AI and MT) was analyzed in a random sample of 17 subjects, demonstrating excellent reliability (kappa = 0.88 based on Cohen’s kappa statistics) [6].

### 2.4. Data Collection

Data collected through chart review included the following variables: age, gender, type of glaucoma, best-corrected visual acuity (VA), intraocular pressure (IOP), medication score, visual field mean deviation (MD) (measured with the central 30–2 program on a Humphrey Visual Field Analyzer, Carl Zeiss Meditec, Dublin, CA, USA), spherical equivalent refractive error (SERE), use of topical FP-agonists or EP2-agonists, SU-PAP grade, intraoperative and postoperative complications, and any additional procedures during follow-up. Decimal VA was converted to the logarithm of the minimum angle of resolution (LogMAR). VA measurements of counting fingers, hand motions, light perception, and no light perception were assigned decimal equivalents of 0.0025, 0.002, 0.0016, and 0.0013, respectively [10]. IOP was assessed using Goldmann applanation tonometry (GAT). The medication score was calculated as one point for each topical medication component or for every 250 mg of oral acetazolamide.

### 2.5. Statistical Analysis

Continuous variables were presented as the mean ± SD, accompanied by 95% confidence intervals (CIs). Pre- and postoperative IOPs, medication scores, and VA were compared across groups categorized by SU-PAP grades using one-way analysis of variance. Survival curve analysis was employed to evaluate successful IOP control, with the uncensored date defined as the earliest occurrence of any of the following: the need for additional glaucoma surgery, less than a 20% reduction in IOP, or postoperative IOP exceeding 18 mmHg (definition A) or 15 mmHg (definition B). Survival rate differences among SU-PAP grades were analyzed using the log-rank test. The Cochran–Armitage trend test was used to compare the proportions of categorical variables across SU-PAP grades. Factors influencing surgical success were identified through a Cox proportional hazards model. All statistical analyses were conducted with JMP Pro version 17.5 (SAS Institute, Inc., Cary, NC, USA). Statistical significance was set at *p* < 0.05. A post hoc power analysis for the log-rank test was carried out using the GofChisquarePower function from the Statsmodels Python package, developed by the Statsmodels Development Team (Open-source community).

## 3. Results

Table 1 summarizes the demographic data of the subjects, including age, gender, preoperative IOP, medication score, visual field MD, and SERE. At the time of surgery, out of 102 eyes, 99 (97%) used topical FP agonists; latanoprost was used in 57% of cases, followed by tafluprost (19%), bimatoprost (17%), and travoprost (7%). Preoperatively, SU-PAP grades were as follows: Grade 0 in 17 eyes (17%), Grade 1 in 42 eyes (41%), Grade 2 in 32 eyes (31%), and Grade 3 in 11 eyes (11%). The proportion of cases with a history of conjunctival manipulation surgery, such as trabeculectomy or EX-PRESS, was 23%.

The IOP and medication score during the follow-up period are summarized in Table 2. Among groups stratified by SU-PAP grade, there were no differences in IOP or medication score between the PAP groups throughout the follow-up period. VA, CECD, and MD were also consistent across the PAP groups during the follow-up period (Table 3). The only exception was that the preoperative CECD was lower in Grade 3.

**Figure 1:**
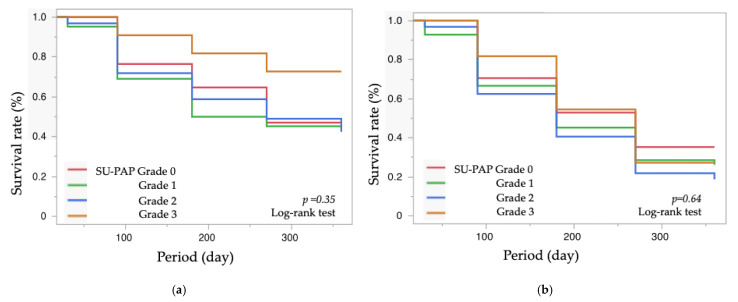
The success rate of IOP control was evaluated for each SU-PAP grade group. In the survival curve analysis, the uncensored date was defined as the earliest occurrence of any of the following: IOP exceeding 18 mmHg (definition A) (**a**) or IOP exceeding 15 mmHg (definition B) (**b**); less than a 20% reduction in IOP; requirement for additional glaucoma surgery; and loss of light perception. Cases that did not meet these criteria by the final visit were treated as censored data.

Survival analysis showed no significant differences in postoperative survival rates between the PAP groups (Figure 1). At 12 months postoperatively, the success rates for Grades 0, 1, 2, and 3 were 47%, 43%, 42%, and 73%, respectively, for definition A (Figure 1a), and 35%, 26%, 19%, and 27%, respectively, for definition B (Figure 1b). A post hoc power analysis for the log-rank test was performed based on the sample sizes (n = 17, 42, 32, 11), survival rates for each group (47%, 43%, 42%, and 73%), and a significance level (α) of 0.05. The resulting power was 88%, indicating a high likelihood of detecting true differences in survival rates among the SU-PAP groups.

The specifics of surgical failures observed in the survival curve analyses are presented in Table 4. No significant differences were found among the SU-PAP grade groups regarding the occurrence of additional glaucoma surgeries, loss of light perception, IOP reduction in less than 20%, or IOP exceeding 15 mmHg or 18 mmHg. The distribution of surgical failure cases appeared consistent across the different SU-PAP grades.

Factors influencing surgical failure were analyzed using a proportional hazards model for both definition A (IOP ≤ 18 mmHg, Table 5) and definition B (IOP ≤ 15 mmHg, Table 6). For definition A, the risk ratio for age (per year) was 0.97, suggesting that older age was significantly associated with a higher likelihood of surgical success (*p* = 0.049). Other factors, including gender, preoperative IOP and medication use, SERE, simultaneous cataract surgery, history of conjunctival manipulation surgeries, and SU-PAP grade, did not show any significant association with surgical failure. For definition B, none of the evaluated variables demonstrated a significant impact on surgical failure.

Finally, surgical complications are shown in Table 7. None of the complications demonstrated a significant difference among the SU-PAP grades. Tube exposure was not recorded in any group.

## 4. Discussion

The survival curve analyses and proportional hazards models revealed no significant differences among SU-PAP grades regarding the effectiveness of AGV in achieving either high-teen IOP levels (Figure 1a, Table 4) or middle-teen IOP levels (Figure 1b, Table 5). Among the various parameters, only older age showed a significant positive correlation with the success rate of AGV in achieving IOP control not exceeding 18 mmHg (Table 5). These results suggest that the severity of PAP may not affect the postoperative outcomes of AGV.

In previous studies, we reported that the severity of PAP worsened the postoperative outcomes of LEC, largely because severe PAP is associated with fibrotic eyelids, which we believe compress the bleb [6,7]. The bleb created by AGV is positioned farther from the limbus compared to LEC, making it less susceptible to eyelid pressure. Long-term use of glaucoma eye drops can cause conjunctival inflammation and fibrosis, negatively affecting the outcomes of LEC [11]. However, in the case of the long-tube surgery, the plate itself functions as a bleb [12], making the bleb less prone to compression and less susceptible to PAP.

The postoperative survival rate was positively correlated with older age. It is well known that younger age negatively impacts the surgical outcomes of tube-shunt surgery [13,14], which is thought to be due to younger individuals having higher wound healing capabilities compared to older individuals [15]. Additionally, as people age, the outflow capacity of both the trabecular and uveoscleral pathways declines [16], contributing to increased IOP in elderly individuals [16,17]. Conversely, aqueous humor production decreases with age [16], as blood supply to the ciliary body is impaired by vascular diseases and inflammation, leading to reduced aqueous humor production [18]. AGV is considered effective for elderly patients with inherently low aqueous humor production, as it lowers IOP by increasing aqueous outflow. While old age is not considered a risk factor for postoperative failure of LEC [19], it is a risk factor for choroidal detachment after LEC [20]. Choroidal detachment, caused by postoperative hypotony [21], has been suggested to induce inflammation, which could potentially lead to bleb failure [20]. Similarly, older age has been reported as a risk factor for choroidal detachment in AGV [18]. However, AGV has a control valve, allowing for better postoperative IOP management compared to LEC [18]. Additionally, as aqueous humor flows through the plate in AGV [12], it is potentially less prone to surgical failure compared to LEC.

LEC is associated with a higher risk of failure in patients with a history of conjunctival incision surgery [22]. However, in this study, such a history did not affect the outcomes of AGV. Due to its structure, where the plate serves as the bleb, AGV is less likely to be influenced by conjunctival fibrosis from previous surgeries compared to LEC. AGV has been proposed as a useful option for eyes in which filtration surgery has failed [1,23], and the results of this study support that proposition. In previous large studies, LEC was favored as the primary surgery, while the Baerveldt Glaucoma Implant (BGI) was more favorable than LEC in cases of unsuccessful LEC and/or pseudophakic eyes [24,25]. Eyes undergoing first-time surgery may have a shorter history of medication use and, therefore, a milder degree of PAP, whereas eyes with a history of previous surgery may exhibit more severe PAP. Thus, part of the discrepancy in previous studies may be explained by differences in PAP severity.

This study has several limitations. The retrospective design may have introduced selection bias. However, the inclusion of all cases meeting the criteria likely reduced bias. Another notable limitation was the lack of detailed medication histories, such as the duration of prostaglandin agonist use and any prior switching between these medications. Patients on long-term use of FP-agonists often have thin, fibrotic conjunctiva as well as PAP. This may increase the risk of device exposure. In addition, PAP can intensify friction between the eyelid and conjunctiva, potentially exacerbating the incidence of exposure during the long-term follow-up. Since this study involves a 12-month follow-up period, a longer observation period is necessary to assess late complications. In subjects with grade 3 SU-PAP, the accuracy of IOP measurements using GAT may have been compromised. In a previous study, we found that grade 3 SU-PAP was associated with overestimation of IOP when measured with GAT compared to rebound tonometry [4]. Therefore, the IOPs reported here may have been higher than the actual values, which could lead to underestimating the IOP-lowering efficacy in the grade 3 group. However, this would not alter the conclusions of this study if other IOP measurement methods were used. We primarily use a combination of aqueous humor suppressants (i.e., β-blockers and carbonic anhydrase inhibitors) as the first choice when topical treatment becomes necessary after glaucoma surgery. This approach may contribute not only to avoiding postoperative inflammation but also to improving pre-existing PAP.

## 5. Conclusions

In summary, the severity of preoperative PAP did not affect the postoperative outcomes of AGV. Additionally, there was no significant difference in outcomes for eyes that had undergone conjunctival manipulation surgery. AGV is a valuable option following LEC failure due to PAP. Moreover, for eyes with severe PAP, where LEC outcomes are expected to be poor, choosing AGV could be a viable option.

## Figures and Tables

**Table 1:** Demographic Data.

Parameters	Mean ± SD or N (%)	95% CI Range or N (%)
N, eyes	102	
Age, years	74.9 ± 7.8	73.4, 76.5
Gender	Male, 55 (54)	Female, 47 (46)
VA, logMAR	0.2 ± 0.4	0.1, 0.3
Preoperative IOP, mmHg	22.8 ± 8.5	24.5, 21.1
Preoperative medication score	3.5 ± 1.1	3.2, 3.7
MD, dB	−17.7 ± 8.5	−19.4, −16.0
SERE, D	−2.4 ± 3.3	−1.8, −3.1
CECD, cells/mm^2^	2103 ± 461	2193, 2012
Prostaglandin use	Yes, 99 (97)	No, 3 (3)
Prostaglandin	Latanoprost, 56 (57)	Tafluprost, 19 (19)
	Bimatoprost, 17 (17)	Travoprost, 7 (7)
SU-PAP grade	Grade 0, 17 (17)	Grade 1, 42 (41)
	Grade 2, 32 (31)	Grade 3, 11 (11)
History of conjunctival manipulation surgery	Yes, 23 (23)	No, 79 (77)

SD, standard deviation; CI, confidence interval; N, number; VA, visual acuity; LogMAR, logarithm of the minimum angle of resolution; IOP, intraocular pressure; MD, mean deviation; dB, decibel; SERE, sphere equivalent refractive error; D, diopter; CECD, corneal endothelial cell density; SU-PAP, Shimane University prostaglandin-associated periorbitopathy grade; AGV, Ahmed glaucoma valve; CE, cataract extraction.

**Table 2:** Comparisons in pre- and post-operative IOP and medication score among groups stratified by SU-PAP grade.

Parameters	Garde 0		Grade 1		Grade 2		Grade 3		*p* Value
	Mean ± SD	95% CI	Mean ± SD	95% CI	Mean ± SD	95% CI	Mean ± SD	95% CI	
IOP, mmHg									
Pre	24.4 ± 9.7	19.4, 29.3	21.8 ± 8.3	19.2, 24.3	21.7 ± 8.3	18.6, 24.8	27.6 ± 5.9	23.7, 31.6	0.15
1M	6.8 ± 3.8	4.8, 8.7	7.6 ± 3.3	6.6, 8.6	8.1 ± 4.1	6.6, 9.6	6.9 ± 2.4	5.3, 8.5	0.59
3M	12.9 ± 8.5	8.6, 17.3	11.2 ± 6.3	9.9, 13.8	12.3 ± 5.6	10.3, 14.3	11.9 ± 4.7	8.8, 15.1	0.94
6M	13.6 ± 4.2	11.5, 15.8	13.8 ± 4.7	12.3, 15.2	12.0 ± 4.0	10.5, 13.4	12.9 ± 4.0	10.2, 15.6	0.35
9M	14.2 ± 5.1	11.6, 16.8	14.0 ± 3.7	12.8, 15.2	12.4 ± 3.8	11.0, 13.8	12.7 ± 3.6	10.3, 15.2	0.27
12M	12.8 ± 3.1	11.2, 14.4	12.9 ± 3.4	11.8, 13.9	12.9 ± 3.3	11.7, 14.1	12.5 ± 2.7	10.7, 14.2	0.98
Medication score									
Pre	3.4 ± 1.0	2.9, 3.8	3.3 ± 1.2	2.9, 3.7	3.6 ± 1.2	3.2, 4.1	3.7 ± 1.0	3.0, 4.4	0.50
1M	0.0 ± 0.0	0.0, 0.0	0.0 ± 0.3	−0.0, 0.1	0.0 ± 0.0	0.0, 0.0	0.0 ± 0.0	0.0, 0.0	0.70
3M	0.3 ± 0.7	−0.1, 0.6	0.4 ± 0.8	0.1, 0.6	0.4 ± 0.8	0.1, 0.7	0.2 ± 0.6	−0.2, 0.6	0.88
6M	1.0 ± 1.2	0.4, 1.6	1.0 ± 1.1	0.6, 1.3	1.2 ± 1.3	0.8, 1.7	0.9 ± 1.0	0.2, 1.6	0.79
9M	1.6 ± 1.1	1.0, 2.2	1.6 ± 1.2	1.3, 2.0	1.6 ± 1.3	1.2, 2.1	1.5 ± 0.8	1.0, 2.1	0.99
12M	1.7 ± 1.1	1.1, 2.3	1.9 ± 1.2	1.6, 2.3	1.9 ± 1.0	1.5, 2.2	2.0 ± 1.0	1.3, 2.7	0.91

*p* values are calculated among SU-PAP groups using one-way analysis of variance (ANOVA). SU-PAP, Shimane University prostaglandin-associated periorbitopathy grade; SD, standard deviation; CI, confidence interval; IOP, intraocular pressure.

**Table 3:** Comparisons in pre- and post-operative VA, CECD, and MD among groups stratified by SU-PAP grade.

Parameters	Garde 0		Grade 1		Grade 2		Grade 3		*p* Value
	Mean ± SD	95% CI	Mean ± SD	95% CI	Mean ± SD	95% CI	Mean ± SD	95% CI	
VA, LogMAR									
Pre	0.3 ± 0.4	0.0, 0.5	0.2 ± 0.4	0.1, 0.3	0.2 ± 0.4	0.1, 0.4	0.3 ± 0.5	−0.1, 0.6	0.81
1M	0.8 ± 1.0	0.2, 1.3	0.7 ± 1.0	0.4, 1.0	0.5 ± 0.8	0.3, 0.8	0.6 ± 0.9	0.0, 1.2	0.86
3M	0.3 ± 0.4	0.1, 0.5	0.3 ± 0.5	0.1, 0.5	0.3 ± 0.4	0.1, 0.4	0.3 ± 0.6	−0.1, 0.7	0.99
6M	0.2 ± 0.4	0.0, 0.4	0.3 ± 0.6	0.1, 0.5	0.3 ± 0.4	0.1, 0.4	0.3 ± 0.6	−0.1, 0.7	0.92
9M	0.2 ± 0.4	0.0, 0.4	0.3 ± 0.6	0.1, 0.5	0.3 ± 0.4	0.1, 0.4	0.3 ± 0.6	−0.1, 0.7	0.96
12M	0.2 ± 0.3	0.0, 0.3	0.3 ± 0.6	0.3, 0.4	0.3 ± 0.4	0.1, 0.4	0.3 ± 0.5	−0.1, 0.6	0.86
CECD, cells/mm^2^									
Pre	2036 ± 523	1822, 2250	2156 ± 456	2014, 2298	2201 ± 244	2113, 2289	1718 ± 679	1262, 2174	0.016 *
12M	2038 ± 496	1783, 2293	2038 ± 499	1883, 2194	2084 ± 393	1940, 2229	1803 ± 801	1265, 2341	0.47
MD, dB									
Pre	−15.9 ± 9.0	−20.5, −11.2	−18.8 ± 8.4	−21.4, −16.2	−16.9 ± 8.7	−20.1, −13.8	−18.9 ± 8.4	−24.5, −13.2	0.60
12M	−15.5 ± 9.2	−20.2, −10.7	−18.4 ± 9.0	−21.3, −15.5	−16.8 ± 8.4	−19.9, −13.6	−19.2 ± 8.5	−24.9, −13.5	0.60

*p* values are calculated among SU-PAP groups using one-way analysis of variance (ANOVA). The * indicates significance levels of 5%. SU-PAP, Shimane University prostaglandin-associated periorbitopathy grade; SD, standard deviation; CI, confidence interval; VA, visual acuity; LogMAR, logarithm of the minimum angle of resolution; CECD, corneal endothelial cell density; MD, mean deviation.

**Table 4:** Reasons for surgical failure in each SU-PAP grade.

Parameter	Garde 0	Grade 1	Grade 2	Grade 3	*p* Value
Additional glaucoma surgery	n (%), 0 (0)	1 (2)	1 (3)	0 (0)	0.82
No light perception	0 (0)	0 (0)	0 (0)	0 (0)	-
IOP reduction less than 20%	7 (41)	21 (50)	16 (50)	1 (9)	0.23
IOP over 18 mmHg	6 (35)	9 (21)	7 (22)	2 (18)	0.33
IOP over 15 mmHg	11 (65)	29 (69)	22 (69)	8 (73)	0.70

*p* values are calculated among SU-PAP groups using the chi-square test. SU-PAP, Shimane university prostaglandin-associated periorbitopathy grade; IOP, intraocular pressure.

**Table 5:** Factors associated with successful IOP control of ≤18 mmHg.

Parameters	Risk Ratio	95% CI	*p* Value
Age, /year	0.97	0.93–1.00	0.049 *
Gender, female/male	1.08	0.59–2.00	0.79
Preoperative IOP, /mmHg	0.98	0.95–1.02	0.40
Preoperative Medication score	1.27	0.99–1.65	0.07
SERE, /D	1.06	0.96–1.18	0.27
History of conjunctival manipulation surgery, y/n	0.70	0.34–1.41	0.31
SU-PAP Grade, 1/0	1.27	0.58–2.78	0.55
SU-PAP Grade, 2/0	1.03	0.46–2.37	0.93
SU-PAP Grade, 3/0	0.49	0.13–1.84	0.29

*p* values are calculated using the Cox proportional hazard model. The * indicates significance levels of 5%. IOP, intraocular pressure; CI, confidence interval; SERE, spherical equivalent refractive error; SU-PAP, Shimane University prostaglandin-associated periorbitopathy grade.

**Table 6:** Factors associated with successful IOP control of ≤15 mmHg.

Parameters	Risk Ratio	95% CI	*p* Value
Age, /year	0.98	0.95–1.01	0.18
Gender, female/male	1.16	0.70–1.93	0.57
Preoperative IOP, /mmHg	1.00	0.97–1.03	0.80
Preoperative Medication score	1.23	0.99–1.54	0.06
SERE, /D	1.02	0.95–1.11	0.63
History of conjunctival manipulation surgery, y/n	0.69	0.38–1.24	0.21
SU-PAP Grade, 1/0	1.41	0.70–2.84	0.34
SU-PAP Grade, 2/0	1.36	0.66–2.86	0.40
SU-PAP Grade, 3/0	1.15	0.46–2.90	0.77

*p* values are calculated using the Cox proportional hazard model. IOP, intraocular pressure; CI, confidence interval; SERE, spherical equivalent refractive error; SU-PAP, Shimane University prostaglandin-associated periorbitopathy grade.

**Table 7:** Recorded surgical complications in each SU-PAP grade.

Parameter	Garde 0	Grade 1	Grade 2	Grade 3	*p* Value
Choroidal detachment	n (%), 0 (0)	4 (4)	3 (3)	3 (3)	0.13
hemorrhagic choroidal detachment	0 (0)	1 (1)	1 (1)	0 (0)	0.84
Scleral infolding	4 (4)	3 (3)	1 (1)	0 (0)	0.05
Retinal edema	0 (0)	0 (0)	1 (1)	0 (0)	0.53
Retinal detachment	0 (0)	1 (1)	0 (0)	0 (0)	0.70

*p* values are calculated among SU-PAP groups using the chi-square test. SU-PAP, Shimane university prostaglandin-associated periorbitopathy grade.

## Data Availability

Data are fully available upon reasonable request to corresponding author.
